# Validity and reliability of the Valkyria Trainer Free^®^ linear position transducer in the propulsive phase of bench press in men

**DOI:** 10.7717/peerj.21357

**Published:** 2026-07-15

**Authors:** Álvaro Huerta Ojeda, Guillermo Barahona-Fuentes, Rodrigo Beltrán-Inostroza, Carlos Jorquera-Aguilera, María-Mercedes Yeomans-Cabrera, Maximiliano Torres-Banduc

**Affiliations:** 1 Núcleo de Investigación en Salud, Actividad Física y Deporte ISAFYD, Universidad de Las Américas, Viña del Mar, Chile; 2 Faculty of Education and Social Sciences, Universidad Nacional Andres Bello, Viña del Mar, Chile; 3 Escuela de Kinesiología, Facultad de Salud y Ciencias Sociales, Universidad de Las Américas, Viña del Mar, Chile; 4 Facultad de Ciencias, Escuela de Nutrición y Dietética, Universidad Mayor, Santiago, Chile; 5 Escuela de Psicología, Facultad de Salud y Ciencias Sociales, Universidad de Las Américas, Santiago, Chile

**Keywords:** Validation study, Reproducibility of results, Equipment and supplies, Muscle contraction, Propulsive phase

## Abstract

**Background:**

Velocity-based training has emerged as a leading approach for quantifying training loads. Therefore, valid and reliable devices must be used to evaluate both movement velocity and power.

**Objective:**

This study determined the validity and reliability of the Valkyria Trainer Free^®^ linear position transducer (VTF^®^ LPT) during the propulsive phase of the bench press in physically healthy individuals.

**Materials and Methods:**

Nineteen physically healthy men participated in the study (22.7 ± 2.1 years). A repeated-measures design was used to compare the inter-day test–retest reliability of velocity and power, while validity was determined by comparing the VTF^®^ LPT with the Chrono-Jump^®^ LPT. The analysis consisted of the intraclass correlation coefficient (ICC), standard error of measurement (SEM), and coefficient of variation (CV) for slow velocity zone: 0.08–0.49 m·s^−1^, power zone: 0.50–0.99 m·s^−1^, and fast velocity zone: 1.00–1.56 m·s^−1^. A CV ≤ 10% and an ICC ≥ 0.80 were considered acceptable reliability, whereas a CV ≤ 5% and an ICC ≥ 0.90 were considered high reliability.

**Results:**

For vertical velocity, the “power zone” and “fast velocity zone” showed a high threshold for CV (≤5%), while the “slow velocity zone” showed an acceptable threshold with a CV of 7.94. For power, the “slow velocity zone” showed an acceptable threshold for CV (≤10%), while the “fast velocity zone” and “power zone” showed a CV equivalent to 10.8 and 4.8%, respectively.

**Conclusion:**

The study’s results demonstrated that VTF^®^ LPT is valid and reliable for assessing velocity and power in physically healthy individuals.

## Introduction

From a functional perspective, focusing on activities of daily living, higher levels of muscle strength are associated with better physical performance (Minematsu et al., 2018). In this sense, it highlights the importance of developing and maintaining high levels of muscle strength, mainly to promote health (D’Onofrio et al., 2023). Furthermore, scientific evidence has shown that muscle strength is not a monolithic quality, but rather a complex ability that manifests itself in different ways (maximum strength, power, velocity, reactivity), and that athletic performance depends on the identification, evaluation, and development of these manifestations through specific training strategies (Huerta Ojeda et al., 2023; Hughes et al., 2023). In this context, the propulsive phase of muscle contraction is critical for assessing neuromuscular potential, allowing us to analyze the individual’s ability to move body mass or lift loads safely, without risk of accidents (Sanchez-Medina, Perez & Gonzalez-Badillo, 2010).

To optimize the assessment and development of muscle strength, it is imperative to have accurate instruments capable of measuring key variables, such as the velocity of movement during the propulsive phase (Jerez-Mayorga et al., 2021; Plakoutsis et al., 2023). This is because the velocity of movement during the propulsive phase in strength exercises with external loads allows different manifestations of muscle strength to be identified. In this context, the analysis of the load-velocity relationship is presented as a valid indicator for defining specific training zones, such as the slow velocity zone, power zone, and fast velocity zone (Bautista et al., 2014), which are associated respectively with the maximum strength zone, the power zone, and the strength-endurance zone (Sanchez-Medina, Perez & Gonzalez-Badillo, 2010). In this sense, devices for assessing the different manifestations of force have evolved from highly sophisticated and stationary equipment (Sørensen et al., 2021) to equipment that allows the reproduction of specific sports gestures under the concept of “controlled natural movement” (Martínez-García et al., 2021).

In this context, during the last decade, velocity-based training (VBT) has led in quantifying training loads (McGrath et al., 2018). In this regard, research has shown that reliability and validity vary considerably across the different commercially available devices (Pérez-Castilla et al., 2019). For example, accelerometers such as Wimu^®^ (Muyor, Granero-Gil & Pino-Ortega, 2018) and mobile applications such as My Jump 2^®^ (Balsalobre-Fernández, Glaister & Lockey, 2015; Bogataj et al., 2020) have proven valid and reliable for measuring linear (vertical) velocity of movement and thereby determining different manifestations of force. However, before implementation, it is recommended that the subjects evaluated have developed adequate technique for the selected exercises and that the devices be used in accordance with the manufacturer’s technical specifications and recommendations. On the other hand, despite the many benefits of these devices—such as their portability, low cost, and ease of access—most provide assessment results as mean values, which means they still rely on wired devices to obtain more accurate or raw data.

In this sense, linear position transducers (LPTs) such as T-Force^®^ (Sousa et al., 2024), GymAware^®^ (Janicijevic et al., 2023), or ChronoJump (Ch-j^®^) (Pérez-Castilla et al., 2019; Sousa et al., 2024) have proven to be valid and reliable for measuring linear velocity. However, the cost of these devices can be prohibitive; therefore, as more affordable options are developed and marketed, it is essential to evaluate their validity and reliability before implementing them in practical or research contexts. Another available LPT on the market is Valkyria Trainer Free^®^ (VTF^®^), developed by IVOLUTION^®^ (Sunchales, Argentina), which can be used on smartphones and tablets, running popular operating systems, such as Android or iOS. In contrast, data acquisition for these devices is done by pairing *via* Bluetooth^®^. It also has a desktop version for Windows^®^ or Mac^®^. In these cases, data acquisition is done through a USB connection to a computer. Compared with traditional systems that require dedicated computers and costly hardware, VTF^®^ is a versatile, mobile-based, and low-cost solution that facilitates access to reliable linear velocity measurements and enables individualized, accurate VBT prescription. However, despite its innovative design and connection versatility, its validity and reliability have not been established. Therefore, the objective of the present study was to determine the validity and reliability of the VTF^®^ LPT during the propulsive phase of the bench press in men. Given this, it was hypothesized that this device provides a reliable and valid method of measuring velocity during the propulsive phase of the bench press in men.

## Materials and Methods

### Research design

**Figure 1:**
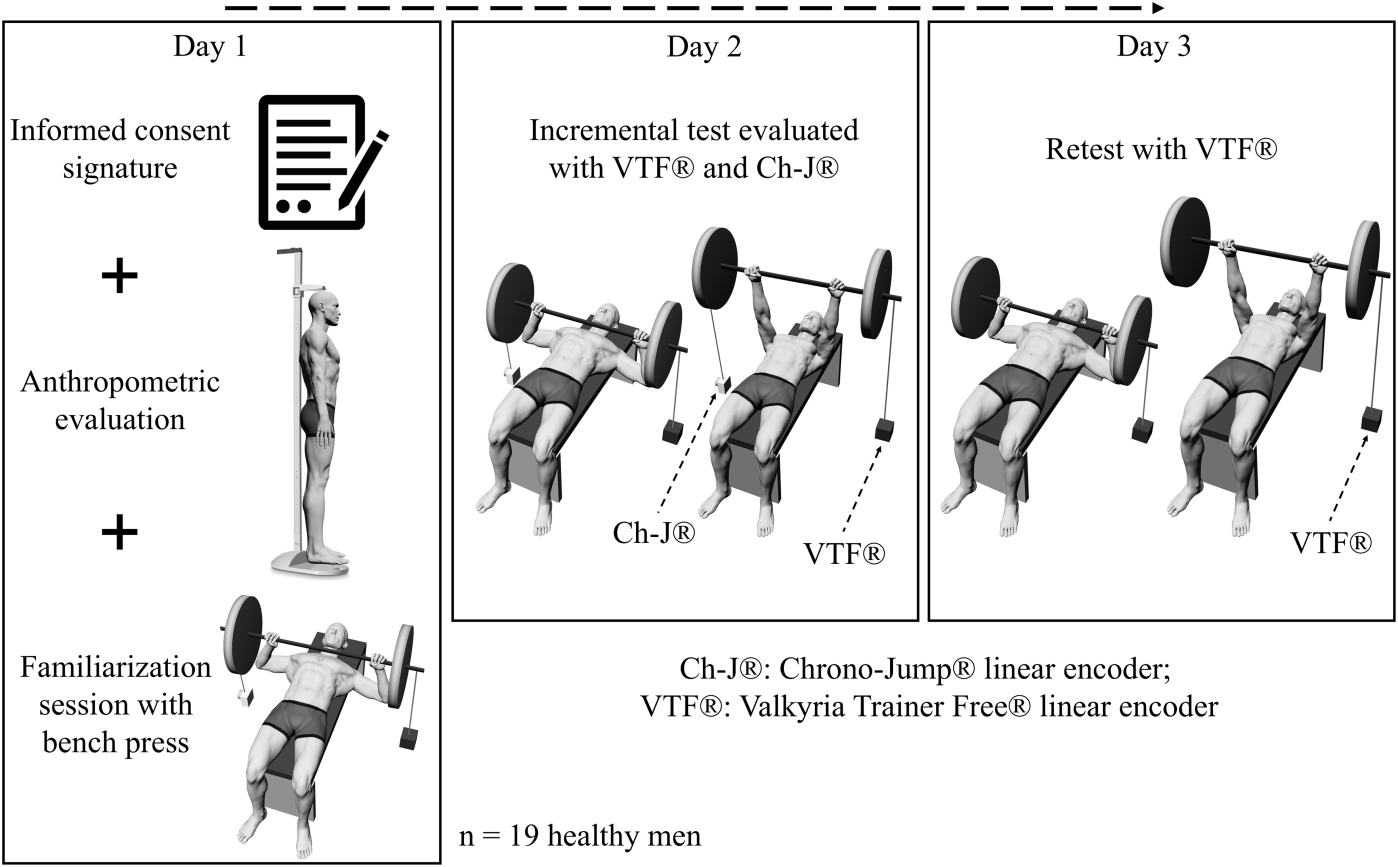
Research design. Ch-J^®^, Chrono-Jump^®^ linear encoder; VTF^®^, Valkyria Trainer Free^®^ linear encoder.

The following study was developed in accordance with the Guidelines for Reporting Reliability and Agreement Studies (GRRAS) (Cuschieri, 2019). A repeated-measures design was used to determine the inter-day test–retest reliability of the mean vertical velocity and mean muscle power of the bench press propulsive phase (Ato, López & Benavente, 2013). Participant recruitment and data collection were conducted between August and November 2024. The participants attended three evaluations at 72-h intervals. During the first session, informed consent was obtained, basic anthropometric assessments were conducted, and participants were familiarized with bench press exercises. During familiarization, the bench press technique was evaluated and, if necessary, corrected and standardized. In the second session, an incremental test was conducted to determine the one-repetition maximum (1RM) for the bench press. In this session, the vertical velocity of execution of all repetitions was recorded simultaneously with a Ch-J^®^ LPT (gold standard) (Pérez-Castilla et al., 2019; Sousa et al., 2024) and VTF^®^ LPT. In the third session, each participant performed three sets: one in the “slow velocity zone,” another in the “power zone,” and a final set in the “fast velocity zone” (the velocities for the three zones are described in the following sections). In this session, the vertical velocity of execution of all repetitions was recorded only with the VTF^®^. To determine inter-day test–retest reliability, the mean vertical velocity of movement and the mean muscle power generated during the propulsive phase of the bench press in this session were compared with the same loads performed in the second session (1RM) (Fig. 1).

### Participants

A preliminary power analysis was conducted using the G*Power software to estimate the required sample size (Faul, 2020). In the absence of directly comparable previous studies reporting effect sizes for the variables and conditions analyzed, the expected effect size was determined based on theoretical assumptions. Data were collected as previously described in Huerta Ojeda et al. (2024). Specifically, tests considered two tails, effect size dz = 0.68, α-error < 0.05, and a power (1-β error) = 0.8. The total sample size was 19 participants.

In this study, nineteen male volunteers were included (Table 1). The participants were recruited from various sports teams at the University of the Americas. Inclusion criteria were as follows: over 18 years of age, strength training experience ≥6 months, specifically bench press experience, physically healthy, and without comorbidities. The exclusion criterion was the presence of locomotor system injuries that prevented bench press exercises. Participant recruitment and evaluations were conducted between September and November 2024. The study’s objectives were stated at the beginning of the research. Before the assessments, the participants accepted the informed consent form submitted *via* Google Forms^®^. The study’s protocol was approved by the Scientific-Ethical Committee of the Universidad de Las Américas, Chile (registration number: CEC_FP_2023011). All study procedures were conducted in accordance with the Declaration of Helsinki (updated in 2013) and the ethical standards for exercise and sports (Harriss, MacSween & Atkinson, 2019).

**Table 1:** Age, anthropometric characteristics, and muscle quality.

	Mean ± SD	Min	Max
Age (years)	22.7 ± 2.1	20.0	27.0
Body mass (kg)	76.4 ± 10.0	63.2	113.6
Height (m)	1.70 ± 0.1	1.63	1.91
BMI (kg/m^2^)	25.2 ± 3.0	22.5	36.7
Fat mass (kg)	13.4 ± 4.1	7.3	27.9
1RM (N)	829.2 ± 150.1	621.0	1,196.2
MQI (1RM/BMI)	3.40 ± 0.5	2.5	4.6

**Note:**

BMI, body mass index; kg, kilograms; kg/m^2^, kilograms per square meter; MQI, muscle quality index; N, Newtons; SD, standard deviation; 1RM, one repetition maximum.

### Characterization of the participants

The characterization included body mass, determined using a Tanita Inner Scan BC-554^®^ digital scale; height, assessed with a stadiometer from the feet to the vertex (Frankfurt plane); body mass index (BMI), calculated using the following equation: BMI = kg/m^2^; and muscle quality index (MQI) obtained using the following equation: MQI = 1RM/BMI.

### Standardized warm-up for bench press evaluations

Participants performed a standardized 20-minute (min) warm-up. The warm-up included the following exercises: 2 × 10-second (s) stretching of the wrist, elbow, and shoulder muscle groups. 1 × 10 repetitions of wrist flexion and extension, 1 × 10 repetitions of flexion and extension of elbows, and 1 × 10 rep flexion, extension, adduction, and abduction of shoulders. Then, 3 × 10 s of the isometric plank with the forearms resting on the floor and the elbows flexed at 90°, 3 × 10 s of the isometric plank with hands resting on the floor and elbows extended at 180°. Finally, participants performed 2.5 repetitions in the bench press, with an intensity between 5–6 on the Borg 1–10 scale (Borg, 1990). To avoid musculoskeletal injuries and to standardize movements, incorrect postures were corrected during familiarization. These corrections also helped the participants focus their efforts on the propulsive phase of the movement, lifting the barbell as quickly as possible.

### Protocol for 1RM assessment

The evaluation of 1RM in the bench press was incremental, starting with only the barbell (20 kg). Then the sets increased by 10 kg. This increase in load was maintained until muscle failure or until the Chrono Jump version 1.4.6.0^®^ software (Barcelona, Spain) projected the participant’s 1RM. Between each set, there was a 3-min pause. During this assessment, participants were asked to perform three repetitions with loads ranging from 20 to 40 kg. As the load increased and depending on the velocity during the propulsive phase recorded by the Ch-J^®^ LPT, participants were asked to perform one or two repetitions at each load. This helped prevent premature fatigue and allowed for the collection of more data for statistical analysis. Based on this data, the load-velocity relationship during the propulsive phase was established for each participant, enabling identification of loads in each velocity zone (“slow velocity zone”, “power zone”, and “fast velocity zone”) (Bautista et al., 2014). In practice, this means that the same absolute load (*e.g*., 40 kg) can place different participants in different velocity zones. Likewise, during the propulsive phase, participants were asked to move the mass vertically as fast as possible. In addition, during the test, participants received verbal encouragement from the research team (McNair et al., 1996). The propulsive phase was recorded simultaneously *via* two LPT systems: (a) a Ch-J^®^ LPT running Chronojump^®^ software version 1.4.6.0 (Barcelona, Spain), used as the gold standard (Pérez-Castilla et al., 2019; Sousa et al., 2024) and (b) a VTF^®^ LPT (Sunchales, Argentina). This last LPT operates with the Valkyria Trainer^®^ version 1.1.9 software (Sunchales, Argentina) for mobile and desktop versions. Although the VTF^®^ LPT system allows raw data extraction, in this validation and reliability study, it was decided to analyze the mean values. This decision is because the Ch-J^®^ LPT device does not provide access to raw data; it applies a digital filter to its data before presenting it in the analysis windows, making it impossible to compare the raw data from both devices. These data are recorded during the propulsive phase, and then the mean value is calculated, equivalent to the mean muscle power. Each LPT was attached to one end of the barbell for simultaneous vertical velocity recording and muscle power calculation in the propulsive phase (Fig. 1). Recording this information enabled analysis of the validity of the VTF^®^ LPT across different loads (20 to 80 kg) and velocity zones (“slow velocity zone,” “power zone,” and “fast velocity zone”) (Bautista et al., 2014). Specifically, data from the three areas were used to evaluate the validity of the VTF^®^ LPT using the “gold standard” method.

### Valkyria Trainer Free^®^ linear position transducer

Regarding the operation of the VTF^®^ LPT, data is read from an incremental rotational encoder sensor mounted on the equipment’s axis (this optical encoder consists of a pair of optoelectronic devices: one is the emitter or light source, and the other is the receiver). This sensor registers 600 pulses per turn and is connected to an “Adafruit nrf52840 feather Express” board, where the information is processed and sent to the software at a sampling frequency of 1 kHz. In turn, the device calculates the mean velocity in the propulsive phase through the following sequence: (a) displacement measurement (calculated according to the number of pulses per revolution of an internal wheel of the LPT), (b) time measurement (time of rotation of the internal wheel of the LPT), (c) calculation of the current velocity (Va = [auxiliary double value − previous displacement]/0.001). These data are recorded during the propulsive phase, and then the mean value, equivalent to the mean linear velocity, is calculated. Subsequently, to calculate the mean power in the propulsive phase of the motion, the following equations are added to the mean velocity calculation: (d) calculation of the current acceleration (Aa = [Va − previous velocity]/0.001), (e) calculation of the current force (Fa = load × [current acceleration + 9.8]), and (f) calculation of the current power (Pa = [Fa × Va]).

### Retest session

In the third evaluation (retest), participants were asked to perform three sets with different loads. The loads were obtained and selected from the 1RM evaluation (session 2). At the same time, the criteria for selecting the three individual loads were the different mean vertical velocities performed in the propulsive phase of the 1RM evaluation. Thus, one set was conducted in the “slow velocity zone,” a zone between 0.08–0.49 m·s^−1^; a second set in the “power zone,” a zone between 0.50–0.99 m·s^−1^; and a third set in “fast velocity zone,” a zone between 1.00–1.59 m·s^−1^ (Bautista et al., 2014). The order of these sets was counterbalanced. Between each set, there was a 3-min pause. In the retest, participants were asked to perform three repetitions at the load assigned for the “fast velocity zone,” two repetitions at the load assigned for the “power zone,” and one repetition at the load assigned for the “slow velocity zone.” Based on these data, the velocity-load relationship for the propulsive phase was established for each participant, allowing the identification of absolute loads for each velocity zone (20 to 80 kg). In practice, this means that within the same velocity zone—for example, the “power zone” (0.50–0.99 m·s^−1^)—each participant corresponds to a different absolute load.

During the propulsive phase, participants were asked to move the mass vertically as fast as possible, and, as in the 1RM assessment, participants received verbal encouragement from the research team (McNair et al., 1996). The values of the mean vertical velocity and the calculation of the mean muscular power in the propulsive phase of the bench press during this session were recorded only with VTF^®^ LPT (Sunchales, Argentina). This information was compared with the VTF^®^ LPT counterpart record obtained during the 1RM evaluation, allowing us to analyze the inter-day test–retest reliability of this new device.

### Data analysis

Mean vertical velocity and mean muscle power from the two devices (both recorded in the propulsive phase of bench press), age, anthropometric parameters, and MQI were classified in a spreadsheet designed for the study. Descriptive data are presented as means and standard deviations (SD). Given 19 participants, the data were normally distributed, as confirmed by the Shapiro–Wilk test (*p* > 0.05). Given that one of the study’s objectives was to analyze the validity of the VTF^®^ LPT and that both LPTs were used simultaneously (one on each side of the bar), all repetitions correctly recorded by both devices during the 1RM assessment were included for this objective (validity). Therefore, although 19 subjects participated, each contributed a different number of repetitions to the dataset. Consequently, the validity analysis was performed using more data points than there were study participants. Then, according to the classification proposed by Bautista et al. (2014), the mean running velocities, along with their corresponding loads (20–80 kg), were categorized into the “slow velocity zone,” “power zone,” and “fast velocity zone,” respectively. These were also analyzed to determine the validity of the VTF^®^ LPT. Absolute reliability was assessed using the standard error of measurement (SEM) and coefficient of variation (CV) to quantify the systematic error. In contrast, relative reliability was evaluated using the intra-class correlation coefficient (ICC), model 3.1 (Koo & Li, 2016). For the reliability analysis, all repetitions correctly recorded by VTF^®^ LPT were included in both the initial test (1RM) and the retest. Therefore, although 19 subjects participated, each participant contributed a different number of repetitions to the dataset. Next, the velocity-load relationship for the propulsive phase was determined for each participant, therefore identifying absolute loads (20 to 80 kg) for each velocity zone. In practice, this means that a single velocity zone, for example, the “slow velocity zone” (0.08–0.49 m·s^−1^), could involve different absolute loads for each participant. All reliability assessments were performed through a customized spreadsheet (Hopkins, 2015). Acceptability cutoffs for the ICC were set at ≥0.80, with ≥0.90 considered high. Acceptable and high thresholds for the CV were set at ≤10% and ≤5%, respectively (James et al., 2017). A 95% confidence interval (95% CI) was used for these analyses. The correlation between the two devices for both mean vertical velocity and mean muscular power during the propulsive phase was calculated using Pearson’s test (Hopkins et al., 2009). The criteria for interpreting the strength of the *r* coefficients were as follows: trivial (<0.1), small (0.1–0.3), moderate (0.3–0.5), high (0.5–0.7), very high (0.7–0.9), or practically perfect (>0.9) (Hopkins et al., 2009). However, Pearson’s correlation cannot detect systematic errors (Weir, 2005). For this reason, systematic bias was examined through Bland–Altman plots (Bland & Altman, 1986). Although this test is initially recommended to analyze the concordance between two procedures, it has also been suggested and is widely used to complement Pearson’s correlation (Weir, 2005). All statistical analyses were performed using Prism version 10.4.1 for Windows^®^. The confidence interval for all statistical analyses was 95% (95% CI), while the significance level for all statistical analyses was *p* < 0.05.

## Results

### Age, anthropometrics, and muscle quality

At the time of the study, the 19 participants were 22.7 ± 2.1 years old, while anthropometric analysis showed a body mass of 76.4 ± 10.0 kg, a height of 1.70 ± 0.1 m, and a BMI of 25.2 ± 3.0 kg/m^2^. Age, anthropometric characteristics, and MQI are reported in Table 1.

### Validation analysis

Mean velocity with loads between 20 and 60 kg demonstrated good absolute reliability (CV ≤ 5%), while mean velocity with 70 (CV = 5.24%) and 80 (CV = 5.13%) kg demonstrated acceptable absolute reliability, while relative reliability was high across all loads (ICC ≥ 0.90). Mean power with loads between 30 and 80 kg demonstrated acceptable absolute reliability (CV ≤ 10%), while relative reliability was high at 30, 50, 60, 70, and 80 kg (ICC ≥ 0.90). The mean values, SD, and 95% confidence intervals are reported in Table 2.

**Table 2:** Validity Valkyria Trainer Free^®^ in propulsive phase (*n* = 19).

Bench press	Ch-J mean ± SD	VTF^®^ mean ± SD	*p*	Δ 95% CI	SEM 95% CI	CV 95% CI	ICC 95% CI
Velocity (m·s^−1^)							
20 kg	1.34 ± 0.13	1.33 ± 0.15	0.692	−0.01 [−0.04 to 0.02]	0.04 [0.03–0.07]	3.36 [2.46–5.31]	0.91 [0.77–0.97]
30 kg	1.15 ± 0.12	1.14 ± 0.13	0.302	−0.01 [−0.03 to 0.01]	0.02 [0.01–0.04]	2.29 [1.66–3.69]	0.96 [0.90–0.98]
40 kg	0.97 ± 0.14	0.96 ± 0.12	0.309	−0.01 [−0.03 to 0.01]	0.03 [0.02–0.05]	3.60 [2.70–5.39]	0.94 [0.86–0.98]
50 kg	0.70 ± 0.17	0.72 ± 0.16	0.010	0.02 [0.00 to 0.03]	0.02 [0.01–0.03]	3.23 [2.42–4.85]	0.98 [0.95–0.99]
60 kg	0.54 ± 0.21	0.54 ± 0.21	0.814	0.00 [−0.01 to 0.01]	0.02 [0.01–0.03]	3.75 [2.83–5.54]	0.99 [0.97–0.99]
70 kg	0.45 ± 0.16	0.46 ± 0.15	0.326	0.01 [−0.01 to 0.02]	0.02 [0.01–0.03]	5.24 [3.80–8.44]	0.98 [0.94–0.99]
80 kg	0.36 ± 0.16	0.37 ± 0.16	0.316	0.01 [−0.01 to 0.02]	0.01 [0.01–0.03]	5.13 [3.53–9.38]	0.99 [0.96–0.99]
Power (W)							
20 kg	422.6 ± 111.8	308.3 ± 51.3	<0.001	−114.2 [−159.4 to −69.0]	57.7 [42.2–91.0]	15.7 [11.5–24.9]	0.59 [0.13–0.84]
30 kg	434.1 ± 96.7	377.0 ± 61.2	<0.001	−57.1 [−79.1 to −34.8]	27.2 [19.7–43.9]	6.7 [4.8–10.8]	0.90 [0.72–0.96]
40 kg	442.7 ± 105.5	406.7 ± 63.4	0.011	−36.0 [−62.7 to −9.3]	36.7 [27.3–55.8]	8.6 [6.4–13.1]	0.84 [0.61–0.93]
50 kg	368.9 ± 118.6	372.6 ± 96.0	0.642	3.7 [−12.8 to 20.3]	23.6 [17.7–35.3]	6.3 [4.7–9.5]	0.95 [0.89–0.98]
60 kg	331.5 ± 141.2	332.6 ± 135.0	0.850	1.1 [−11.5 to 13.8]	18.6 [14.0–27.5]	5.6 [4.2–8.2]	0.98 [0.95–0.99]
70 kg	319.4 ± 120.9	325.8 ± 114.4	0.359	6.4 [−8.1 to 21.0]	17.9 [12.9–28.8]	5.5 [4.0–8.9]	0.98 [0.94–0.99]
80 kg	292.3 ± 136.6	299.1 ± 136.9	0.357	6.8 [−9.0 to 22.6]	15.6 [10.7–28.6]	5.3 [3.6–9.6]	0.99 [0.96–0.99]

**Note:**

Ch-J, Chronojump^®^ linear position transductor (encoder); CI, confidence interval; ICC, intra-class correlation coefficient; CV, coefficient of variation; kg, kilograms; m·s^−1^, meters per second; *p*, *p*-value; SD, standard deviation; SEM, standard error of the mean; VTF, Valkyria Trainer Free^®^ linear position transductor (encoder); W, watts; Δ, variation delta.

In parallel, the analysis by velocity zones between the two devices shows that the “power zone” and “fast velocity zone” have high CV thresholds (≤5%). In contrast, the “slow velocity zone” has an acceptable threshold with a CV of 7.94. In the analysis of the ICC by velocity zones between the two devices, the three zones show a high threshold (≥0.90). When analyzing the mean muscle power corresponding to the velocity zones between both devices, it is observed that the “slow velocity zone” presents an acceptable threshold for the CV (≤10%), while the “fast velocity zone” and “power zone” present a CV equivalent to 10.8% and 4.8%, respectively. In the analysis of the ICC for the mean muscle power corresponding to the velocity zones between both devices, the “slow velocity zone” and “power zone” present a high threshold (≥0.90), while the “fast velocity zone” presents an ICC equivalent to 0.79. The mean values, SD, and 95% confidence intervals are reported in Table 3.

**Table 3:** Validity Valkyria Trainer Free^®^ in propulsive phase (*n* = 19).

Bench press	Ch-J mean ± SD	VTF^®^ mean ± SD	*p*	Δ 95% CI	SEM 95% CI	CV 95% CI	ICC 95% CI
Velocity (m·s^−1^)							
*0.08–0.49 m·s^−1^	0.31 ± 0.11	0.33 ± 0.10	0.005	0.02 [0.00–0.03]	0.02 [0.02–0.03]	7.94 [6.34–10.61]	0.94 (0.89–0.97]
**0.50–0.99 m·s^−1^	0.71 ± 0.14	0.72 ± 0.15	0.217	0.01 [0.00–0.01]	0.02 [0.01–0.02]	3.26 [2.70–4.12]	0.97 [0.95–0.98]
***1.00–1.56 m·s^−1^	1.23 ± 0.14	1.21 ± 0.16	0.111	−0.02 [−0.02 to 0.00]	0.03 [0.02–0.04]	2.70 [2.17–3.55]	0.95 [0.91–0.97]
Power (W)							
*0.08–0.49 m·s^−1^	229.5 ± 80.0	245.8 ± 75.6	0.006	16.2 [4.9–27.7]	21.8 [17.4–29.1]	9.1 [7.3–12.2]	0.92 [0.85–0.96]
**0.50–0.99 m·s^−1^	394.2 ± 92.0	394.3 ± 83.2	0.983	−1.6 [−9.8 to 6.1]	19.2 [15.9–24.3]	4.8 [4.0–6.1]	0.95 [0.91–0.97]
***1.00–1.56 m·s^−1^	461.6 ± 108.0	374.8 ± 84.0	<0.000	−83.2 [−105.8 to −61.2]	45.2 [36.4–59.5]	10.8 [8.7–14.2]	0.79 [0.62–0.88]

**Note:**

Ch-J, Chronojump^®^ linear position transductor (encoder); CI, confidence interval; ICC, intra-class correlation coefficient; CV, coefficient of variation; kg, kilograms; m·s^−1^, meters per second; *p*, *p*-value; SD, standard deviation; SEM, standard error of the mean; VTF, Valkyria Trainer Free^®^ linear position transductor (encoder); W, watts; Δ, variation delta; *, slow velocity zone; **, power zone; ***, fast velocity zone.

The correlation analysis showed that the mean vertical velocity evaluated through both LPTs presented a practically perfect *r* coefficient (*r* = 0.99, 95% CI [0.99–0.99], *r*^2^ = 0.98, *p* < 0.0001). On the other hand, the Bland–Altman analysis shows a common bias of −0.003 ± 0.042 m·s^−1^ (95% limits of agreement from −0.088 to 0.080). While the mean muscle power in the propulsive phase, calculated by both devices, presents a very high *r* coefficient (*r* = 0.89, 95% CI [0.80–0.94], *r*^2^ = 0.80, *p* < 0.0001). Finally, the Bland–Altman analysis shows a common bias of 22.97 ± 61.59 W (95% limits of agreement from −97.73 to 143.79).

### Reliability analysis

The analysis of velocity zones between the initial test and the retest shows that the “slow velocity zone” and “fast velocity zone” have high CV thresholds (>5%), whereas the “power zone” has an acceptable CV of 5.92. In the ICC analysis velocity zones, the “slow velocity zone” and the “power zone” exhibit a high threshold (≥0.90). However, the “fast velocity zone” has an ICC of 0.76. When analyzing the mean muscle power corresponding to the velocity zones between the test and retest, it is observed that the “slow velocity zone” and “fast velocity zone” have high CV thresholds (≤5%), while the “power zone” has an acceptable threshold, with a CV of 6.08. In the ICC analysis for mean muscle power, all three zones exhibit a high threshold (≥0.90). The mean values, standard deviation, and 95% confidence intervals are presented in Table 4.

**Table 4:** Reliability test–retest Valkyria Trainer Free^®^ in propulsive phase (*n* = 19).

Bench press	Test mean ± SD	Retest mean ± SD	*p*	Δ 95% CI	SEM 95% CI	CV 95% CI	ICC 95% CI
Velocity (m·s^−1^)							
*0.08–0.49 m·s^−1^	0.45 ± 0.02	0.48 ± 0.01	0.013	0.02 [0.00–0.04]	0.00 [0.00–0.02]	2.08 [1.25–5.99]	0.94 [0.55–0.99]
**0.50–0.99 m·s^−1^	0.71 ± 0.14	0.71 ± 0.16	0.821	0 [−0.02 to 0.02]	0.04 [0.03–0.06]	5.92 [4.55–8.46]	0.93 [0.84–0.97]
***1.00–1.56 m·s^−1^	1.10 ± 0.06	1.08 ± 0.05	0.293	−0.01 [−0.04 to 0.01]	0.03 [0.02–0.05]	2.85 [1.99–5.01]	0.76 [0.33–0.93]
Power (W)							
*0.08–0.49 m·s^−1^	319.70 ± 38.66	325.8 ± 32.88	0.563	6.10 [−20.84 to 33.04]	15.34 [9.19–44.09]	4.75 [2.84–13.66]	0.95 [0.62–0.99]
**0.50–0.99 m·s^−1^	375.74 ± 77.81	381.28 ± 70.22	0.434	5.53 [−8.91 to 19.98]	23.04 [17.72–32.92]	6.08 [4.68–8.69]	0.91 [0.80–0.96]
***1.00–1.56 m·s^−1^	403.19 ± 73.16	394.97 ± 69.94	0.243	−8.22 [−22.89 to 6.45]	16.33 [11.57–27.73]	4.09 [2.89–6.94]	0.95 [0.86–0.98]

**Note:**

CI, confidence interval; ICC, intra-class correlation coefficient; CV, coefficient of variation; kg, kilograms; m·s^−1^, meters per second; *p*, *p*-value; SD, standard deviation; SEM, standard error of the mean; W, watts; Δ, variation delta; *, slow velocity zone; **, power zone; ***, fast velocity zone.

When evaluating the agreement of the VTF^®^ LPT interday, the mean vertical velocities associated with the different loads used in the bench press show non-significant differences between the test and retest (*p* > 0.05). While the 30, 40, 50, and 80 kg loads present a high threshold for CV (≤5%), and the 60 and 70 kg loads have an acceptable threshold for this variable (CV ≤ 10%). In the analysis of the ICC for mean vertical velocity in bench press between tests, the 40, 50, and 80 kg loads present a high threshold (≥0.90), the 60 and 70 kg loads present an acceptable threshold (≥0.80), and the 30 kg load presents an ICC equivalent to 0.40. When analyzing mean muscle power during bench press between tests, no significant differences were observed at any load (*p* > 0.05). While the 40, 50, 70, and 80 kg loads present a high threshold for CV (≤5%), the 30 and 60 kg loads present a CV equivalent to 5.2% and 8.0%, respectively. In the analysis of the ICC for the mean muscular power in bench press between tests, the 40, 50, 60, 70, and 80 kg loads show high ICCs (≥0.90), whereas the 30 kg load shows an ICC of 0.42. Despite this last indicator, the values observed in this analysis suggest that the VTF^®^ LPT has a low systematic error in estimating the mean vertical velocity during the propulsive phase of the interday bench press. The mean values, SDs, and 95% confidence intervals of the reliability analysis are reported in Table 5.

**Table 5:** Reliability test–retest Valkyria Trainer Free^®^ in propulsive phase (*n* = 19).

Bench press	Test mean ± SD	Retest mean ± SD	*p*	Δ 95% CI	SEM 95% CI	CV 95% CI	ICC 95% CI
Velocity (m·s^−1^)							
30 kg	1.10 ± 0.07	1.08 ± 0.04	0.540	−0.01 [−0.07 to 0.04]	0.05 [0.03–0.10]	4.70 [3.11–9.58]	0.40 [−0.36 to 0.84]
40 kg	0.98 ± 0.11	0.96 ± 0.13	0.555	−0.01 [−0.07 to 0.04]	0.03 [0.02–0.09]	3.89 [2.43–9.55]	0.96 [0.75–0.99]
50 kg	0.75 ± 0.14	0.76 ± 0.15	0.425	0.01 [−0.02 to 0.04]	0.03 [0.02–0.06]	4.41 [2.98–8.46]	0.96 [0.85–0.99]
60 kg	0.62 ± 0.13	0.62 ± 0.10	0.979	0.00 [−0.05 to 0.05]	0.04 [0.03–0.09]	7.60 [5.02–15.48]	0.88 [0.53–0.97]
70 kg	0.48 ± 0.04	0.48 ± 0.04	0.642	0.00 [−0.03 to 0.05]	0.02 [0.01–0.07]	5.20 [3.11–14.96]	0.81 [0.02–0.97]
80 kg	0.58 ± 0.17	0.48 ± 0.16	0.053	−0.09 [−0.20 to 0.00]	0.00 [0.00–0.26]	1.54 [0.69–49.39]	0.99 [−0.41 to 0.99]
Power (W)							
30 kg	354.9 ± 26.8	352.4 ± 19.1	0.779	−2.5 [−22.6 to 17.5]	18.5 [12.5–35.4]	5.2 [3.5–10.0]	0.42 [−0.28 to 0.83]
40 kg	417.7 ± 58.5	410.5 ± 63.3	0.516	−7.2 [−33.9 to 19.4]	17.9 [11.2–44.1]	4.3 [2.7–10.6]	0.96 [0.76–0.99]
50 kg	381.1 ± 90.3	394.7 ± 89.8	0.148	13.6 [−6.0 to 33.3]	18.12 [12.2–34.7]	4.6 [3.1–8.9]	0.97 [0.88–0.99]
60 kg	368.8 ± 92.9	366.4 ± 68.8	0.878	−2.3 [−37.5 to 32.7]	29.72 [19.6–60.5]	8.0 [5.3–16.4]	0.91 [0.62–0.98]
70 kg	334.2 ± 27.1	335.4 ± 28.3	0.901	1.2 [−23.9 to 26.3]	14.3 [8.5–41.2]	4.2 [2.5–12.3]	0.90 [0.34–0.98]
80 kg	475.2 ± 146.7	393.9 ± 132.1	0.080	−81.3 [−212.6 to 49.9]	10.3 [4.6–329.7]	2.3 [1.0–75.8]	0.98 [−0.68 to 0.99]

**Note:**

CI, confidence interval; ICC, intra-class correlation coefficient; CV, coefficient of variation; kg, kilograms; m·s^−1^, meters per second; *p*, *p*-value; SD, standard deviation; SEM, standard error of the mean; W, watts; Δ, variation delta.

**Figure 2:**
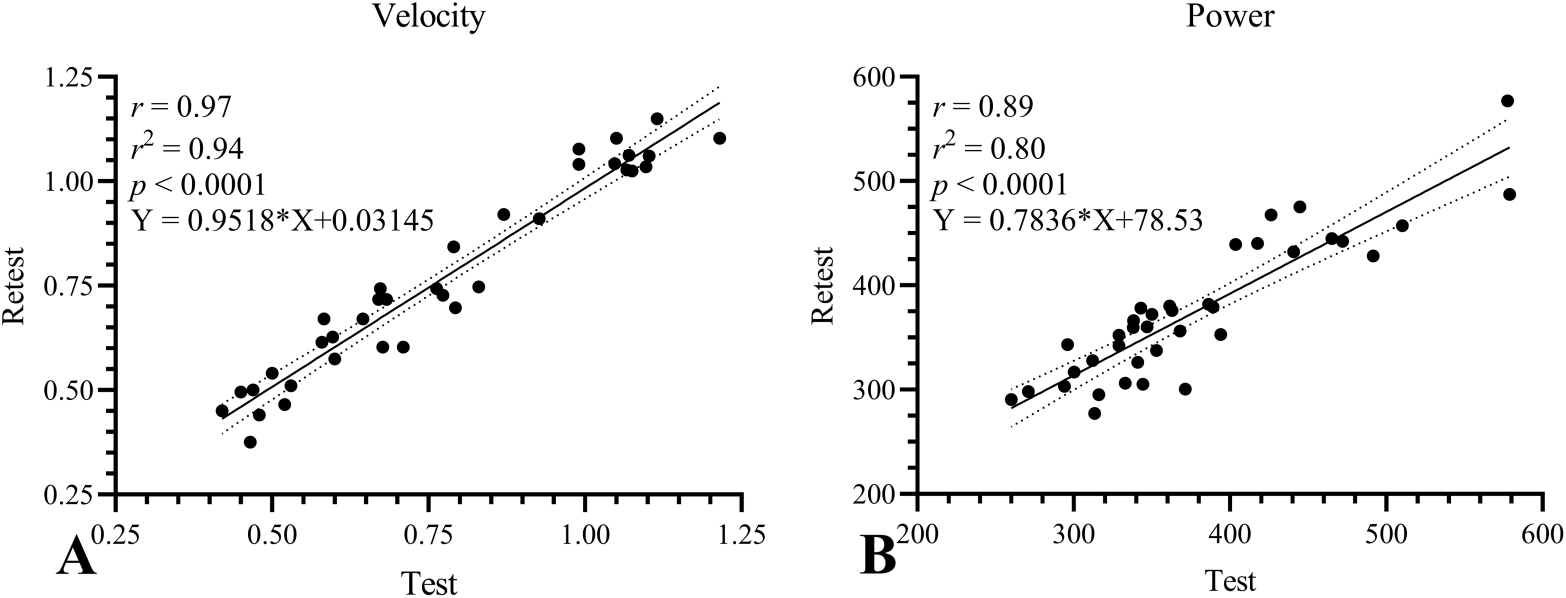
Regression analysis for mean vertical velocity and mean muscular power in the propulsive phase in bench presses between test–retest. (A) Velocity; (B) power.

The correlation analysis showed that the mean vertical velocity evaluated using the VTF^®^ LPT across tests had a practically perfect *r* coefficient (*r* = 0.97). In contrast, the mean muscle power, calculated from the mean vertical velocity, between tests showed a very high *r* coefficient (*r* = 0.89). The graphs of the correlations and the regression lines, with their respective confidence intervals, are shown in Fig. 2.

**Figure 3:**
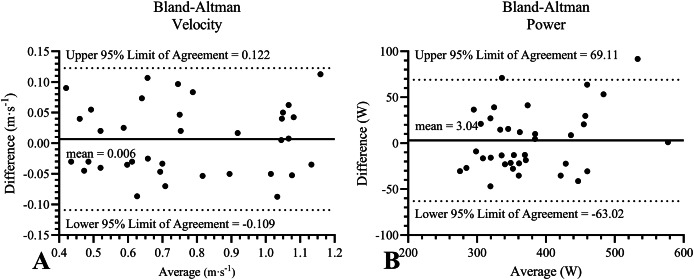
Bland–Altman analysis. The solid line represents the average of the differences between variables evaluated through the Valkyria Trainer Free^®^ in test–retest (*n* repetitios = 38). The segmented lines represent 95% of the upper and lower confidence limits. m·s^−1^, meters per second; N, Newton. (A) Velocity; (B) power.

When comparing the mean values and differences of mean vertical velocity at test and retest, the Bland–Altman analysis showed a common bias of 0.006 ± 0.059 m·s^−1^ (95% limits of agreement from −0.109 to 0.122). In parallel, when comparing the mean values and differences of mean muscular power at the test and retest, the Bland–Altman analysis showed a common bias of 3.04 ± 33.71 W (95% limits of agreement from −63.02 to 69.11). The values of the common bias and 95% upper and lower confidence limits for the different window lengths are reported in Fig. 3.

## Discussion

At the end of the study, which aimed to determine the validity and reliability of the VTF^®^ LPT during the propulsive phase of the bench press in physically healthy individuals, the results showed that the VTF^®^ LPT is valid, with high inter-day agreement and reproducibility. Specifically, the VTF^®^ LPT is valid and reliable for evaluating the mean linear velocity of movement (m·s^−1^) and the mean muscular power (W) during the propulsive phase of the bench press, supporting the research hypothesis.

### Mean linear velocity of movement

The velocity at which a specific sporting gesture or movement is performed, and therefore, quantifying the training load based on that execution velocity constitutes the foundation of VBT (McGrath et al., 2018). For this reason, there is a need to develop valid, reliable, and low-cost devices to evaluate the linear velocity of movements and muscle power, mainly in the propulsive phase (Sanchez-Medina, Perez & Gonzalez-Badillo, 2010). In this context, the VTF^®^ LPT showed high agreement in the three velocity zones when compared to Ch-J^®^ LPT (slow velocity zone: CV = 7.94% and ICC = 0.94; power zone: CV = 3.26% and ICC = 0.97; and fast velocity zone: CV = 2.70% and ICC = 0.95), providing a valid and reliable alternative for evaluating the linear velocity of movements. In parallel, other devices on the market for measuring muscle strength in the field have proven valid and reliable for assessing the linear velocity of movements (Balsalobre-Fernández, Glaister & Lockey, 2015; Bogataj et al., 2020; Janicijevic et al., 2023; Muyor, Granero-Gil & Pino-Ortega, 2018; Pérez-Castilla et al., 2019; Sousa et al., 2024). For example, GymAware^®^ and T-Force^®^ (Janicijevic et al., 2023). However, although fairly constant, there is a systematic bias between the two devices in measuring the magnitude of the maximum linear velocity (Janicijevic et al., 2023). Along the same lines, the results of the validity and reliability analysis of the Vitruve^®^ LPT suggest that this device should only be used under the following conditions: (a) mean linear velocities lower than 0.75 m·s^−1^ in the bench press and (b) mean linear velocities lower than 0.45 m·s^−1^ in the half squat. These suggestions are based on findings observed at higher velocities, particularly the low agreement between devices for both exercises (Janicijevic et al., 2023). Also, a recent study concluded that some commercially available LTPs may be inadequate to objectively monitor the full spectrum of the load-velocity curve (González-Galán et al., 2024). This background indicates that existing devices on the market have varying levels of accuracy; therefore, only some would allow accurate VBT quantification (González-Galán et al., 2024; Janicijevic et al., 2023; Pérez-Castilla et al., 2019). In another experience, mobile applications have also demonstrated the ability to measure linear (vertical) velocity of motion (Bogataj et al., 2020) and, therefore, are among the devices available for VBTs. However, to the best of our knowledge, mobile applications only provide access to the “summary data” of the replays, preventing exploration of the “raw data” (Balsalobre-Fernández, Glaister & Lockey, 2015; Bogataj et al., 2020) and limiting their versatility for accurate load prescription in VBTs. In contrast, the VTF^®^ LPT has demonstrated good versatility for mobile and laptop applications, connecting *via* Bluetooth or USB. Access to these connections allows users to obtain “summary data” or “raw data,” depending on the requirements of the evaluations or training. Based on the described background and the results of the present study, the VTF^®^ LPT emerges as a valid and reliable tool for assessing the mean linear velocity of movements. However, when analyzing the mean velocity during the propulsive phase, it should be noted that the device exhibited lower reliability in the “fast velocity zone.” The lower reliability observed in this zone (1.00–1.56 m·s^−1^) can be explained by the biomechanical characteristics inherent in high-velocity movements. In this zone, although the coefficient of variation was relatively low (CV = 2.85), the ICC showed more moderate values (ICC = 0.76), and this trend was even more evident when analyzing light absolute loads, such as 30 kg (CV = 4.70; ICC = 0.40). One possible explanation is that at higher execution velocities, the propulsive phase of the movement is considerably shorter, reducing the time window available to accurately record the displacement and velocity of the barbell (García-Ramos & Weakley, 2021). In this context, small variations in form from one repetition to the next can lead to relatively large changes in the recorded velocity values. Furthermore, the use of light loads—typically associated with this velocity range—can cause greater barbell oscillations and trajectory variations, thereby increasing the variability of the signal captured by devices based on linear position transducers (Banyard, Nosaka & Haff, 2017; Weakley et al., 2021). Taken together, more explosive movements, shorter movement duration, and greater sensitivity of the measurement system to small mechanical variations could explain the reduced reliability observed in this velocity range.

### Mean power of the propulsive phase

In parallel, based on equations and proper programming, LPT allows the estimation of force, rate of force development (RFD), power, rate of power development (RPD), and other variables during linear movements. These magnitudes can be calculated in linear movements’ eccentric and concentric (propulsive) phases. Also, the mean and peak values of all the magnitudes described can be obtained. During the present study, because it is considered a critical component of neuromuscular potential (Sanchez-Medina, Perez & Gonzalez-Badillo, 2010), the validity and reliability of mean muscle power in the propulsive phase of the bench press were determined. In this context, the VTF^®^ LPT showed that both the mean muscle power developed in the “slow velocity zone” and “power zone” were within the acceptable CV and ICC thresholds (CV ≤ 10% and ICC ≥ 0.90). Similarly to the analysis of mean propulsive velocity, the “fast velocity zone” showed a CV and an ICC outside the acceptable range (CV = 10.8% and ICC = 0.79). In the first perspective of analysis, most research on the validity and reliability of LPTs focuses on mean and maximum linear velocities, excluding differences in force (González-Galán et al., 2024; Janicijevic et al., 2023; Muyor, Granero-Gil & Pino-Ortega, 2018; Pérez-Castilla et al., 2019; Sousa et al., 2024). Therefore, it is difficult to compare the results of the present study with similar investigations. Then, understanding that the results of the different magnitudes of force are based on an equation that includes as a variable the velocity recorded by the LPT (m·s^−1^) and the mass (kg) programmed by the evaluator, it is understandable that all the investigations consulted have focused their attention on the vertical velocity of the movements since any discordance in these velocities should have repercussions on the estimates of the different magnitudes of force. In this sense, evidence shows that light loads executed in the fast velocity zone exhibit higher CV and lower ICC (Koo & Li, 2016), demonstrating that, for light loads, there is less agreement between devices. During the execution of the light loads in the “fast velocity zone” developed in the present study, the barbell was asymmetrically lifted. Also, the light loads allow for greater barbell travel and, therefore, greater data recording by the LPTs. Both the asymmetric execution of the bench press and the longer barbell stroke with light loads could alter the amount of data recorded by both the Ch-J^®^ and VTF^®^, differentiating the powers calculated by both LPT (*r* = 0.89). When analyzing the inter-day agreement (test–retest) of the VTF^®^ LPTht load of 30 kg light load shows CV and ICC values outside the acceptable thresholds. Since power is calculated as the product of force and velocity, small variations in velocity measurements—especially during fast movements with light loads—can be amplified in the final power calculation, increasing measurement variability and potentially explaining the lower reliability values observed with light loads. Nevertheless, the VTF^®^ LPT emerges as a valid and reliable tool for assessing muscle power during the propulsive phase of the bench press.

### Limitations

To determine the validity and reliability of the mean vertical velocity of movement and the mean muscular power in the propulsive phase, the mean values of both variables were obtained from both Ch-J^®^ and VTF^®^. In this case, the values observed in the windows of the two software allow access to different force-derived magnitudes. However, the Ch-J^®^ LPT does not allow raw data extraction, while the VTF^®^ LPT presents only two variables in the raw data (force and time). This prevented a comparative analysis of both LPTs from the raw data. Finally, given the scope of the present investigation, the results only allow us to ensure the validity and reliability of the VTF^®^ LPT for mean velocity and mean power in the propulsive (concentric) phase. Consequently, the peak values and the other force magnitudes need further exploration.

## Conclusions

Considering the results of inter-device agreement, it is concluded that the VTF^®^ LPT is valid for assessing mean linear (vertical) velocity of movements and mean muscle power in the propulsive phase in physically healthy individuals. Likewise, based on the high inter-day agreement and reproducibility, it is concluded that the VTF^®^ LPT is reliable for assessing both mean velocity and mean power in physically healthy individuals. These results allow us to accept the research hypothesis.

### Practical applications

Due to its mass, device connectivity (Bluetooth or USB), and portability, the VTF^®^ PLT enables valid, reliable field measurements. Also, the possibility of using mobile applications to connect the VTF^®^ PLT allows for the different needs of evaluation and VBT.

## Supplemental Information

10.7717/peerj.21357/supp-1 Supplemental Information 1 Raw data.

10.7717/peerj.21357/supp-2 Supplemental Information 2 STROBE Checklist.

## Additional Information and Declarations

### Data Availability

The following information was supplied regarding data availability:

The raw data is available in the Supplemental File and at figshare: Huerta, Álvaro (2025). Dataset. figshare. Dataset. https://doi.org/10.6084/m9.figshare.30964303.v1.
