# Supplementary material for: Validity and reliability of the Valkyria Trainer Free® linear position transducer in the propulsive phase of bench press in men
Source: PeerJ. 2026 Jul 15;14:e21357. doi: 10.7717/peerj.21357 (PMC13380235; doi:10.7717/peerj.21357)
Supplement: Supplemental Information 2 [file peerj-14-21357-s002.docx]

STROBE Statement—checklist of items that should be included in reports of observational studies

|  | Item No. | Recommendation | Page  No. | Relevant text from manuscript |
| --- | --- | --- | --- | --- |
| **Title and abstract** | 1 | (a) Indicate the study’s design with a commonly used term in the title or the abstract | 3 | Title: "Validity and reliability of the Valkyria Trainer Free®..."  Abstract: "A repeated-measures design was used to compare the inter-day test-retest reliability..." |
|  |  | (b) Provide in the abstract an informative and balanced summary of what was done and what was found | 3 | "Background... Objective... Materials and methods... Results... Conclusion." |
| Introduction | | | |  |
| Background/rationale | 2 | Explain the scientific background and rationale for the investigation being reported | 4-5 | "Therefore, valid and reliable devices must be used to evaluate both movement velocity and power." ... "However, the cost of these devices can be prohibitive... it is essential to evaluate their validity and reliability before implementing them..." |
| Objectives | 3 | State specific objectives, including any prespecified hypotheses | 5 | "Therefore, the objective of the present study was to determine the validity and reliability of the VTF® LPT... Given this, it was hypothesized that this device provides a reliable and valid method..." |
| Methods | | | |  |
| Study design | 4 | Present key elements of study design early in the paper | 5 | "A repeated-measures design was used to determine the inter-day test-retest reliability of the bench press propulsive phase..." |
| Setting | 5 | Describe the setting, locations, and relevant dates, including periods of recruitment, exposure, follow-up, and data collection | 5-6 | "Participant recruitment and data collection were conducted between August and November 2024." ... "The participants attended three evaluations at 72-hour intervals." |
| Participants | 6 | (a) Cohort study—Give the eligibility criteria, and the sources and methods of selection of participants. Describe methods of follow-up  Case-control study—Give the eligibility criteria, and the sources and methods of case ascertainment and control selection. Give the rationale for the choice of cases and controls  Cross-sectional study—Give the eligibility criteria, and the sources and methods of selection of participants | 5-6 | "Inclusion criteria were as follows: over 18 years of age, strength training experience ≥ 6 months... The exclusion criterion was the presence of locomotor system injuries..." |
|  |  | (b) Cohort study—For matched studies, give matching criteria and number of exposed and unexposed  Case-control study—For matched studies, give matching criteria and the number of controls per case | N/A | Not applicable (Repeated measures design). |
| Variables | 7 | Clearly define all outcomes, exposures, predictors, potential confounders, and effect modifiers. Give diagnostic criteria, if applicable | 5,7 | Outcomes: "mean vertical velocity of movement and the mean muscle power generated during the propulsive phase."    Zones: "slow velocity zone... power zone... and fast velocity zone" |
| Data sources/ measurement | 8* | For each variable of interest, give sources of data and details of methods of assessment (measurement). Describe comparability of assessment methods if there is more than one group | 6-7 | "The propulsive phase was recorded simultaneously via two LPT systems: (a) a Ch-J® LPT... and (b) a VTF® LPT..." |
| Bias | 9 | Describe any efforts to address potential sources of bias | 6, 7 | "The order of these sets was counterbalanced." (to avoid fatigue/order bias).  "...participants were asked to move the mass vertically as fast as possible, and... received verbal encouragement from the research team" (to standardize effort). |
| Study size | 10 | Explain how the study size was arrived at | 5 | "Statistical software (G*Power...) was used to calculate the sample... The total sample size was 19 participants." |

Continued on next page

| Quantitative variables | 11 | Explain how quantitative variables were handled in the analyses. If applicable, describe which groupings were chosen and why | 7 | "velocity zone: 0.08-0.49 m·s⁻¹, power zone: 0.50-0.99 m·s⁻¹, and fast velocity zone: 1.00-1.56 m·s⁻¹." |
| --- | --- | --- | --- | --- |
| Statistical methods | 12 | (a) Describe all statistical methods, including those used to control for confounding | 7-8 | "Shapiro-Wilk test... intraclass correlation coefficient (ICC), standard error of measurement (SEM), and coefficient of variation (CV)... Pearson's test... Bland-Altman plots" |
|  |  | (b) Describe any methods used to examine subgroups and interactions | 7 | Analysis performed by "velocity zones" and by "loads between 20 and 60 kg" vs "70 and 80 kg". |
|  |  | (c) Explain how missing data were addressed | N/A | Not explicitly stated (data appears complete for n=19). |
|  |  | (d) Cohort study—If applicable, explain how loss to follow-up was addressed  Case-control study—If applicable, explain how matching of cases and controls was addressed  Cross-sectional study—If applicable, describe analytical methods taking account of sampling strategy | N/A | Not applicable. |
|  |  | (e) Describe any sensitivity analyses | N/A | Not explicitly stated. |
| Results | | | | |
| Participants | 13* | (a) Report numbers of individuals at each stage of study—eg numbers potentially eligible, examined for eligibility, confirmed eligible, included in the study, completing follow-up, and analysed | 5 | "The total sample size was 19 participants." "In this study, nineteen male volunteers were included". (Manuscript does not report number of screened vs excluded candidates, only the final n). |
|  |  | (b) Give reasons for non-participation at each stage | N/A | No information available in text regarding excluded participants count. |
|  |  | (c) Consider use of a flow diagram | not applicable | Figure 1 shows the research design flow, but not a participant recruitment flow diagram. |
| Descriptive data | 14* | (a) Give characteristics of study participants (eg demographic, clinical, social) and information on exposures and potential confounders | 8 | "At the time of the study, the 19 participants were 22.7 ± 2.1 years old... body mass of 76.4 ± 10.0 kg..." (Full details in Table 1). |
|  |  | (b) Indicate number of participants with missing data for each variable of interest | N/A | Not applicable/Not reported. |
|  |  | (c) Cohort study—Summarise follow-up time (eg, average and total amount) | N/A | Not applicable/Not reported. |
| Outcome data | 15* | Cohort study—Report numbers of outcome events or summary measures over time | N/A | Not applicable/Not reported. |
|  |  | Case-control study—Report numbers in each exposure category, or summary measures of exposure | N/A | Not applicable/Not reported. |
|  |  | Cross-sectional study—Report numbers of outcome events or summary measures | 8-9 | Detailed summary measures (Mean ± SD) for Velocity and Power are reported in text and Tables 2, 3, and 4. |
| Main results | 16 | (a) Give unadjusted estimates and, if applicable, confounder-adjusted estimates and their precision (eg, 95% confidence interval). Make clear which confounders were adjusted for and why they were included | 8-9 | "Mean velocity... demonstrated good absolute reliability (CV ≤ 5%)... relative reliability was high across all loads (ICC ≥ 0.90)." 95% CI reported in Tables. |
|  |  | (b) Report category boundaries when continuous variables were categorized | 7 | Categories defined as: "slow velocity zone... power zone... fast velocity zone" |
|  |  | (c) If relevant, consider translating estimates of relative risk into absolute risk for a meaningful time period | N/A | Not applicable/Not reported. |

Continued on next page

| Other analyses | 17 | Report other analyses done—eg analyses of subgroups and interactions, and sensitivity analyses | 8-9 | Bland-Altman analysis reported: "common bias of -0.003 ± 0.042 m·s⁻¹...". Analysis separated by load (kg) and velocity zones. |
| --- | --- | --- | --- | --- |
| Discussion | | | | |
| Key results | 18 | Summarise key results with reference to study objectives | 10 | "the results showed that the VTF® LPT is valid, with high inter-day agreement and reproducibility." |
| Limitations | 19 | Discuss limitations of the study, taking into account sources of potential bias or imprecision. Discuss both direction and magnitude of any potential bias | 11 | "However, the Ch-J® LPT does not allow raw data extraction... This prevented a comparative analysis of both LPTs from the raw data." |
| Interpretation | 20 | Give a cautious overall interpretation of results considering objectives, limitations, multiplicity of analyses, results from similar studies, and other relevant evidence | 10-11 | "Based on the described background and the results of the present study, the VTB® LPT emerges as a valid and reliable tool..." Compares with GymAware and Vitruve results. |
| Generalisability | 21 | Discuss the generalisability (external validity) of the study results | 11-12 | "Due to its mass, device connectivity... and portability, the VTF® PLT enables valid, reliable field measurements." (Limited to "physically healthy individuals" as stated in Conclusions). |
| Other information | |  | | |
| Funding | 22 | Give the source of funding and the role of the funders for the present study and, if applicable, for the original study on which the present article is based | 12 | "The authors received no funding for this work." / "This research received no external funding." |

*Give information separately for cases and controls in case-control studies and, if applicable, for exposed and unexposed groups in cohort and cross-sectional studies.

**Note:** An Explanation and Elaboration article discusses each checklist item and gives methodological background and published examples of transparent reporting. The STROBE checklist is best used in conjunction with this article (freely available on the Web sites of PLoS Medicine at http://www.plosmedicine.org/, Annals of Internal Medicine at http://www.annals.org/, and Epidemiology at http://www.epidem.com/). Information on the STROBE Initiative is available at www.strobe-statement.org.
